# The growth of the mastoid volume in children with a cochlear implant

**DOI:** 10.1038/s41598-023-37160-7

**Published:** 2023-07-06

**Authors:** Minna Tirkkonen, Matti Iso-Mustajärvi, Anandhan Dhanasingh, Pia Linder, Katariina Myller, Aarno Dietz

**Affiliations:** 1 Department of Otorhinolaryngology, Institute of Clinical Medicine, University of Eastern Finland, Kuopio, Finland; 2 Department of Otorhinolaryngology, Kuopio University Hospital, Kuopio, Finland; 3 Head of Translational Science Communication, MED-EL, Innsbruck, Austria; 4 Department of Oncology, Vaasa Central Hospital, Vaasa, Finland; 5 Department of Radiology, Turku University Hospital, Turku, Finland

**Keywords:** Cochlea, Inner ear, Computed tomography

## Abstract

The aim of this study was to understand the mastoid volume development in children who undergo cochlear implantation surgery. Cochlear implant (CI) database of our clinic (Kuopio University Hospital) was reviewed for computed tomography (CT) images of CI patients (age under 12 years at the time of implantation) with a minimum time interval of twelve months between their pre- and postoperative CT. Eight patients (nine ears) were found eligible for inclusion. Three linear measurements were taken by using picture archiving and communication systems (PACS) software and the volume of the MACS was measured with Seg 3D software. The mastoid volume increased on average 817.5 mm^3^ between the pre- and the postoperative imaging time point. The linear distances measured between anatomical points like the round window (RW)- bony ear canal (BEC), the RW-sigmoid sinus (SS), the BEC-SS, and the mastoid tip (MT)-superior semicircular canal (SSC) increased significantly with the age of the patient at both the pre-op and post-op time points. The linear measurements between key anatomical points and mastoid volume showed a positive linear correlation. The correlation between linear measurement and volume were significant between the MT-SSC (*r* = 0.706, *p* = 0.002), RW-SS (*r* = 0.646, *p* = 0.005) and RW-BEC (*r* = 0.646, *p* = 0.005). Based on our findings from the CI implanted patients and comparing it with the previous literature findings from non-CI implanted patients, we could say that the CI surgery seem to have no effect on the development of mastoid volume in children.

## Introduction

Surgical implantation of a cochlear implant (CI) has become a standard treatment option in restoring hearing loss in patients of every age group, including new-born infants as young as 9–10 months old^1^. After placing the electrode array optimally inside the scala-tympani (ST) compartment of the cochlea, the excess electrode lead is coiled in the mastoid drilled cavity following a standard surgical procedure^2^. The literature shows a natural growth in the overall mastoid thickness as measured by the linear distance between the cochlear entrance and the skull surface as well the overall width of the skull as measured from one side to the other with age. There is a rapid growth in mastoid thickness until approximately 4 years of age reaching on average of 27 mm after which the growth slows down and reaches a plateau of 30–35 mm around puberty^3^. A similar growth pattern is seen in the mastoid with a surface area of around 3.5–4 cm^2^ at the age of one and an average growth rate of 1–1.2 cm^2^ per year up to 6 years of age. Thereafter, the growth rate decreases and reaches a maximum surface area of 12 cm^2^ at puberty^4^.

Literature has shown that electrode array extrusion out of the cochlea may occur following CI surgery at a rate of 2–3% with the straight lateral wall electrode type compared to < 1% with the pre-curved electrode type^5–8^. While the rate of electrode array extrusion is much less with the pre-curved electrode type, there are other complications like electrode tip fold-over and scalar deviation is reported to have higher rate with the pre-curved electrode type than the straight electrode type^8^. While it is a logical thinking that excess electrode lead in the mastoid drilled cavity would relax in response to the natural growth of mastoid with time, but this alone cannot be the primary reason for the electrode array extrusion. Recently Alhabib et al. reported change in electrode lead coiled configuration in the mastoid drilled cavity in response to mastoid growth in 8 subjects out of 14 subjects studied, but there was no extrusion of the electrode array^9^. The etiology behind the electrode array extrusion/migration is not fully explained. The growth of the mastoid area is proposed as one factor affecting its migration by pulling on the excess lead and consequently the electrode gradually out of the cochlea. The excess coiling of the electrode lead stores kinetic energy which always wants to escape and if the electrode lead is not adequately secured, it could spring back pulling the electrode array out of the cochlea. While there are surgical and device solution available to secure the excess electrode lead in the mastoid drilled cavity, electrode array migration is still reported at a smaller rate.

There are currently no studies reporting the effect of CI surgery on the development of mastoid size/volume and thus the possibility of growth as a factor contributing to electrode migration. The primary aim of this retrospective study was to understand the development of mastoid size/volume in children who underwent cochlear implantation at our centre and to detect any long-term complications in the middle ear or mastoid following CI surgery. The secondary aim was to standardize the method of measuring the mastoid size and correlate it with patient age.

## Methods

This study has been conducted according to the Finnish legislation and it has been granted the institutional approval by Kuopio University Hospital (No. 5551864). This study is retrospective in nature and all patients included have been treated according to the institution’s guidelines at the time of treatment. No additional treatment or diagnostics were conducted to perform this study. Therefore, Kuopio University Hospital has waived the need for informed consent for the study. Selection of subjects were based on medical reports of all CI patients under the age of twelve who had preoperative computed tomography (CT) image of the temporal bone and postoperative CT reimaging after a minimum of one year in the Kuopio University Hospital, Finland. Patients with pathologies in middle ear and mastoid were excluded from the study.

### Software tool

**Figure 1:**
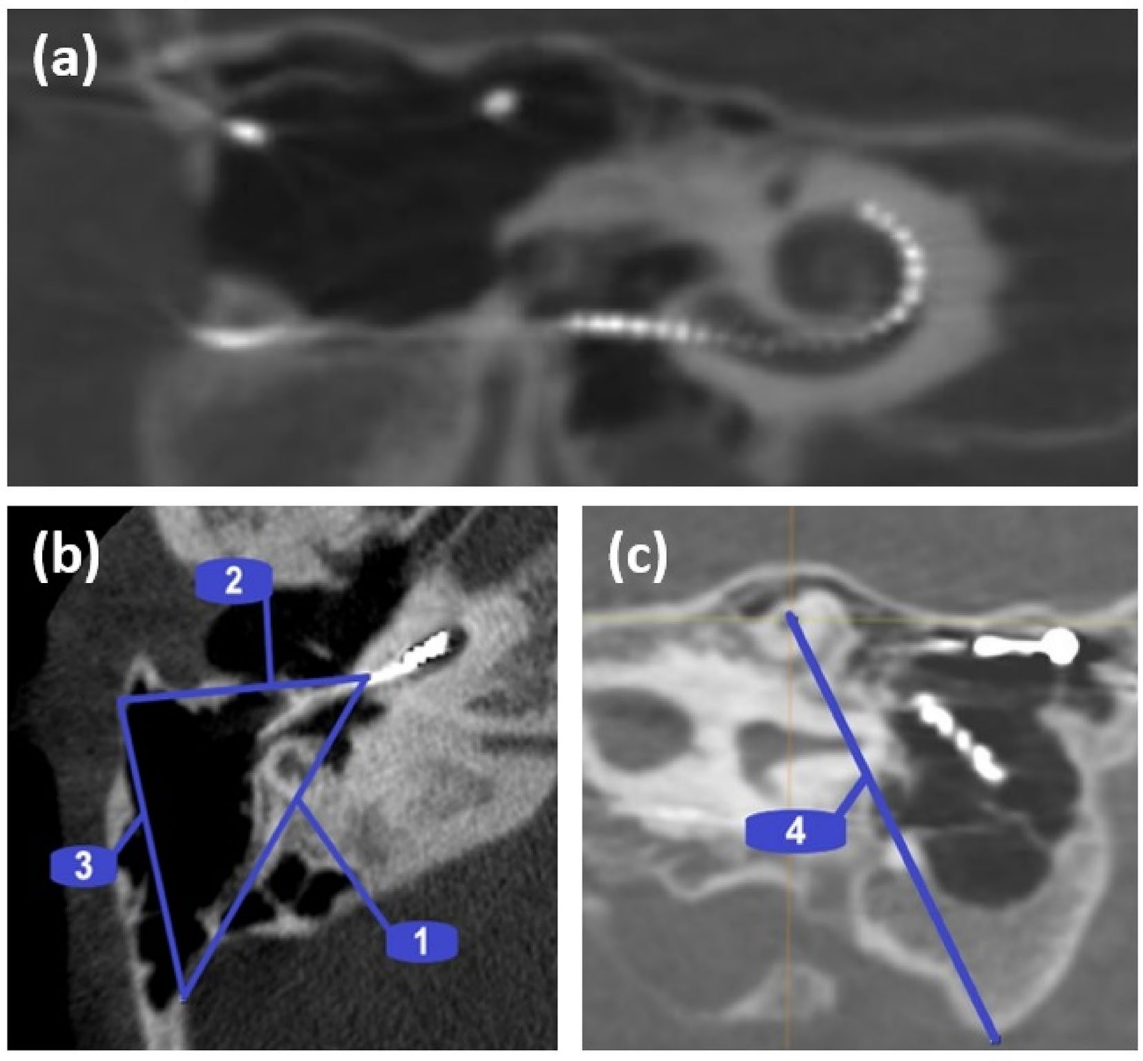
Assessment of the mastoid size and volume. Assessment starts from the cochlear view in the oblique coronal plane (**a**). The measurement lines 1, 2, and 3 are drawn in the axial view. Line 1 represents the measurement between the round window (RW) and the sigmoid sinus (SS), line 2 represents the measurement between the RW and the bony ear canal (BEC), and line 3 represents the measurement between the BEC and the SS (**b**). To capture the mastoid volume, line 4 goes in the z-direction between the superior semicircular canal (SSC) and the mastoid tip (MT) (**c**).

Picture archiving and communication systems (PACS) software utilizing multiplanar reconstruction (MPR) was used to standardize the measurements plane for each patient. Firstly, the cochlear view as described by Xu et al.^10^ in the oblique coronal plane was applied in MPR to define a consistent plane for the measurements (Fig. 1a). The axial plane corresponding to the cochlear view was used to capture the growth in the mastoid process. Three measurements were taken by forming a triangle in the axial plane (Fig. 1b): the first line was drawn from the round window (RW) entrance to the mastoid edge following the surface of the sigmoid sinus (SS), the second line from the RW to the mastoid edge following the surface of the bony ear canal (BEC) and the third line from the lateral surface of the SS to the posterolateral part of the BEC. The lateral surface of SS was used as measuring point instead of cortex because the cortex might be drilled out during the CI-surgery. Finally, the thickness was measured by identifying the tip of the superior semicircular canal (SSC) and the most inferior part of the mastoid tip (MT) (Fig. 1c). The same measurements were then taken from the postoperative images. Linear distances between MT and SSC, RW and BEC, RW and SS, BEC and SS were compared between the pre- and postoperative images to evaluate mastoid growth. Overall growth was calculated to be the difference between volume measurements during the follow-up period.

**Figure 2:**
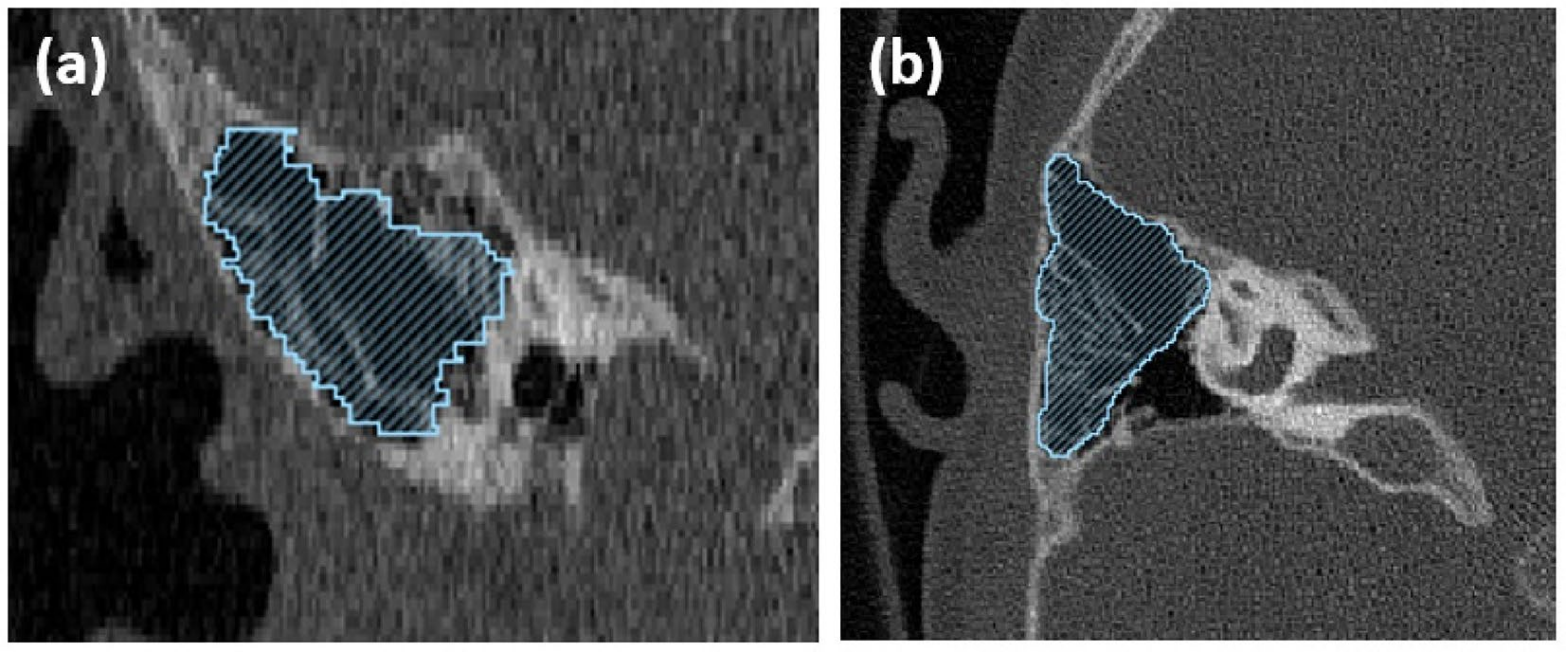
Calculation of mastoid volume using Seg 3D. Blue shaded area represents the mastoid in both coronal (**a**) and axial view (**b**).

The mastoid volume was measured with Seg 3D software (Version 2.4.4, 2016, Volumetric Image Segmentation and Visualization, Scientific Computing and Imaging Institute, University of Utah, USA, https://www.sci.utah.edu/cibc-software/seg3d.html) after visualizing the boundaries of the mastoid air cells manually on every section. The software measures the volume and reports in both pixels and cubic millimetres. A demonstration of volume measurements is presented in Fig. 2 showing in both axial and coronal plane. The growth of the mastoid was evaluated from the change in volume between the preoperative CT images taken before the first implantation and the postoperative images.

### Statistical analyses

Data analyses were performed with Statistical Packages for the Social Sciences (SPSS) version 27 (SPSS Inc., Chicago, IL, USA, https://www.ibm.com/products/spss-statistics) software. Wilcoxon signed-rank test was used for statistical significance testing and Pearson’s test was used for correlation testing. A *p*-value ≤ 0.05 was considered statistically significant and correlation value of *r* > 0.6 were considered strong.

## Results

In preliminary search, we found 20 patients (14 females and 6 males) that fulfilled the criterion. 12 patients (8 females and 4 males) had to be excluded from the study for the following reasons: the imaging area did not meet our region of interests, the resolution of the CT image was inadequate for measurements, postoperative field of images did not contain the whole mastoid area, or the preoperative imaging was performed so long ago that they were not available in digital form. Ultimately, eight patients (one bilaterally implanted, thus nine ears) (6 females and 2 males) were eligible for inclusion in this study. The patient details are given in Table 1. Unfortunately, for patient 3 the image quality of the preoperative images was insufficient for volume measurements and therefore excluded from that analysis. None of these 8 patients have any history of malnutrition, growth retardation disorders, bone diseases, temporal bone fracture, cholesteatoma, chronic suppurative otitis media or revised mastoid surgeries.

**Table 1:** General information on the age at CT imaging, follow-up times and indications for re-imaging.

Patient	Age at implantation (months) and side	Electrode array	Time between pre- and postoperative imaging (months)	Age at postoperative imaging (months)	Indication for reimaging
1	11 (left)	SSE	28	39	Suspected electrode migration (right)
1	11 (right)	SSE	28	39	Suspected electrode migration
2	15 (right)	CA	69	81	Routine protocol
3	15 (left)	CA	113	112	Routine protocol
4	12 (right)	CA	51	63	Routine protocol
5	19 (right)	CA	89	107	Routine protocol
6	26 (right)	CA	87	111	Routine protocol
7	47 (right)	CA	89	136	Preoperative for implantation on the left side
8	36 (right)	CA	82	118	Routine protocol

*SSE* slim straight electrode, *CA* contour advance.

The shortest interval between pre- and post-operative CT imaging was 28 months and the longest was 113 months (Table 1). The average time was 76 months. The volume of the mastoid increased on average 817.5 mm^3^ (minimum: − 7 mm^3^ and maximum: 1639 mm^3^) between the pre-, and the post-operative imaging time points (Table 2).

**Table 2:** Mastoid volume (mm^3^) measurement from both pre- and postoperative images.

Patient	Pre-op volume (mm^3^)	Post-op volume (mm^3^)	Growth (mm^3^)	Follow-up time (months)	Growth per year (mm^3^)
1(r)	390	1525	+ 1135	28	487.12
1(l)	370	1538	+ 1168	28	501.28
2	1289	1708	+ 419	69	72.87
3	n/a	2806	n/a	113	n/a
4	984	1849	+ 865	51	203.53
5	2235	2915	+ 680	89	91.64
6	1735	3374	+ 1639	87	226.07
7	2500	2493	− 7	89	No growth
8	3419	4060	+ 641	82	93.85

*r* right ear, *l* left ear, *n/a* not applicable.

**Figure 3:**
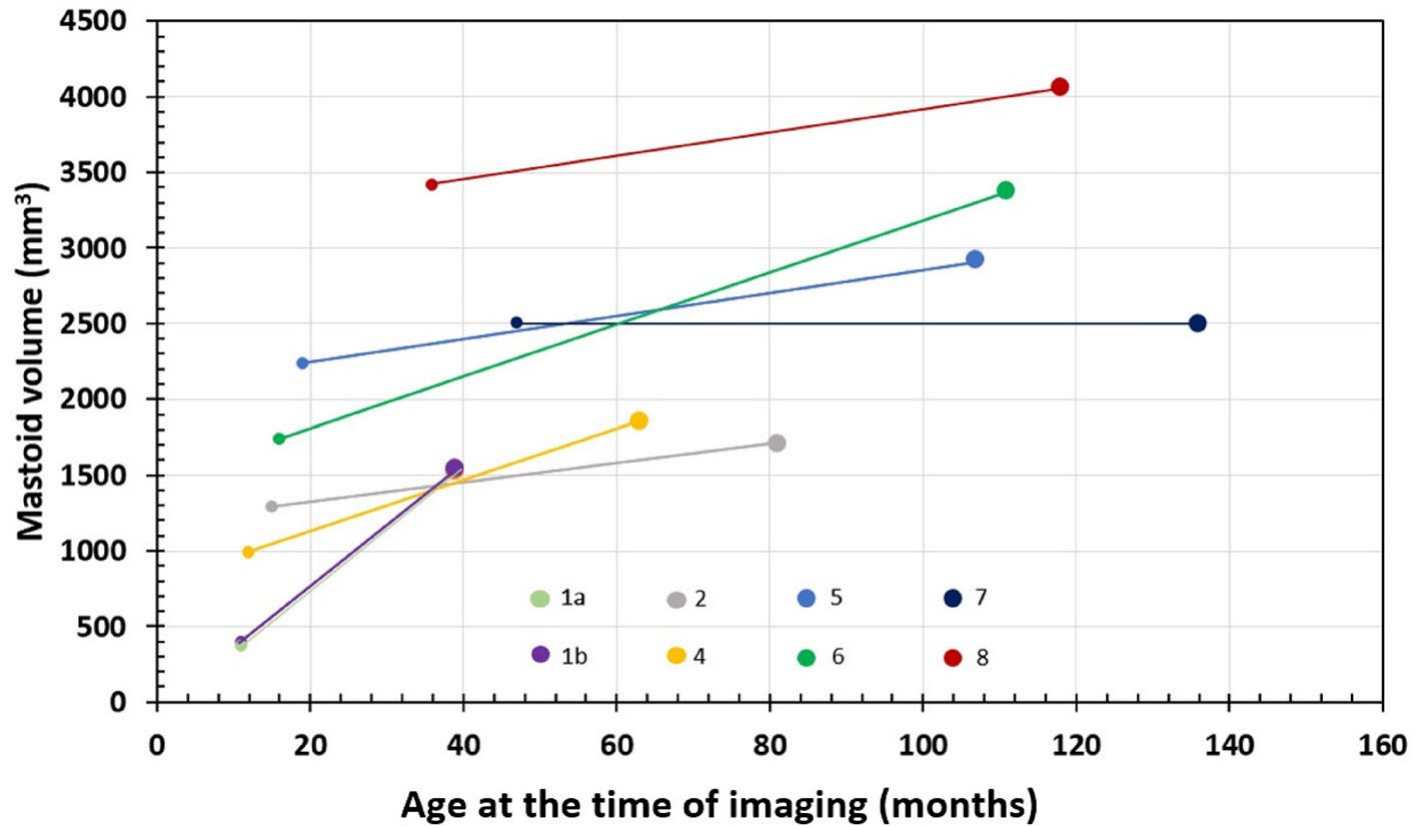
Change in mastoid volume during the follow-up time.

Figure 3 shows the increase in mastoid volume over time for every patient except patient 3. The overall change in the mastoid volume during the follow-up time was statistically significant (*p* < 0.05).

Within the follow-up time, there was a growth in the linear distance between the anatomical points measured from the pre-, and the post-operative images. The values for all 8 patients are given in Table 3. There was a statistically significant difference in the linear measurements regarding the growth between the follow-up scans RW to BEC (*p* < 0.05), MT to SSC (*p* < 0.05) and RW to SS (*p* < 0.05). There was no statistical significance in growth during the follow-up measured between the scans in SS and BEC measure (*p* = 0.23).

**Table 3:** Linear measurements between the different anatomical points measured from pre-, and post-operative images to evaluate growth in mastoid over time.

Patient	MT to SSC (mm)			RW to BEC (mm)			RW to SS (mm)			BEC to SS (mm)			Volume (mm^3^)	Time (month)
	Pre-op	Post-op	Growth	Pre-op	Post-op	Growth	Pre-op	Post-op	Growth	Pre-op	Post-op	Growth	Overall Growth	Follow-up
1 (r)	21.0	28.3	+ 7.3	15.7	15.6	− 0.1	20.4	21.6	+ 1.2	10.5	11.8	+ 1.3	+ 1135	28
1 (l)	19.3	27.3	+ 8.0	14.6	15.7	+ 1.1	22.5	25.1	+ 2.6	13.1	14.2	+ 1.1	+ 1168	28
2	25.3	32.1	+ 6.8	18.9	19.2	+ 0.3	24.3	26.0	+ 1.7	15.7	15.9	+ 0.2	+ 419	69
3	23.2	34.1	+ 10.9	13.9	18.3	+ 4.4	21.7	24.1	+ 2.4	12.9	13.2	+ 0.3	n/a	113
4	20.2	30.2	+ 10.0	10.5	18.6	+ 8.1	18.1	23.0	+ 4.9	13.4	11.9	− 1.5	+ 865	51
5	24.9	31.4	+ 6.5	17.7	18.8	+ 1.1	23.6	31.4	+ 7.8	14.9	25.1	+ 10.2	+ 680	89
6	23.4	31.0	+ 7.6	16.5	17.1	+ 0.6	22.6	23.4	+ 0.8	16.8	18.4	+ 1.6	+ 1639	87
7	32.5	38.5	+ 6.0	17.8	20.0	+ 2.2	27.4	28.5	+ 1.1	15.1	13.8	− 1.3	− 7	89
8	28.1	32.3	+ 4.2	18.8	20.6	+ 1.8	26.1	27.9	+ 1.8	14.1	14.5	+ 0.4	+ 641	82
Mean	24.2	31.7	+ 7.5	16.0	18.2	+ 2.2	23.0	25.7	+ 2.7	14.1	13.9	+ 1.4	+ 727	76
Min	19.3	27.3	+ 4.2	10.5	15.6	− 0.1	18.1	21.6	+ 0.8	10.5	11.8	− 1.5	− 7	28
Max	32.5	38.5	+ 10.9	18.9	20.6	+ 8.1	27.4	31.4	+ 7.8	16.8	25.1	+ 10.2	+ 1639	113

*l* left, *r* right, *MT* mastoid tip, *SSC* superior semicircular canal, *RW* round window, *BEC* bony ear canal, *SS* sigmoid sinus. Overall growth: change of volume between the follow-up scans.

**Figure 4:**
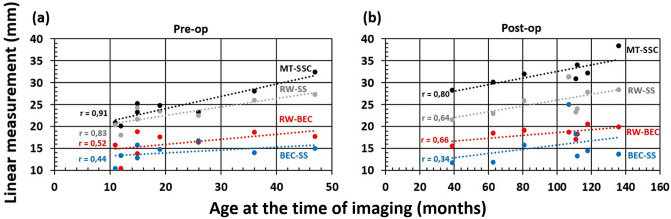
Correlation plots between linear measurements and age at preoperative time point (**a**), and postoperative time point (**b**).

The linear measurements (MT-SSC; RW-SS; RW-BEC; BEC-SS) were found to increase linearly with the age of patients as measured at both pre- and postoperative as shown in Fig. 4a,b.

**Figure 5:**
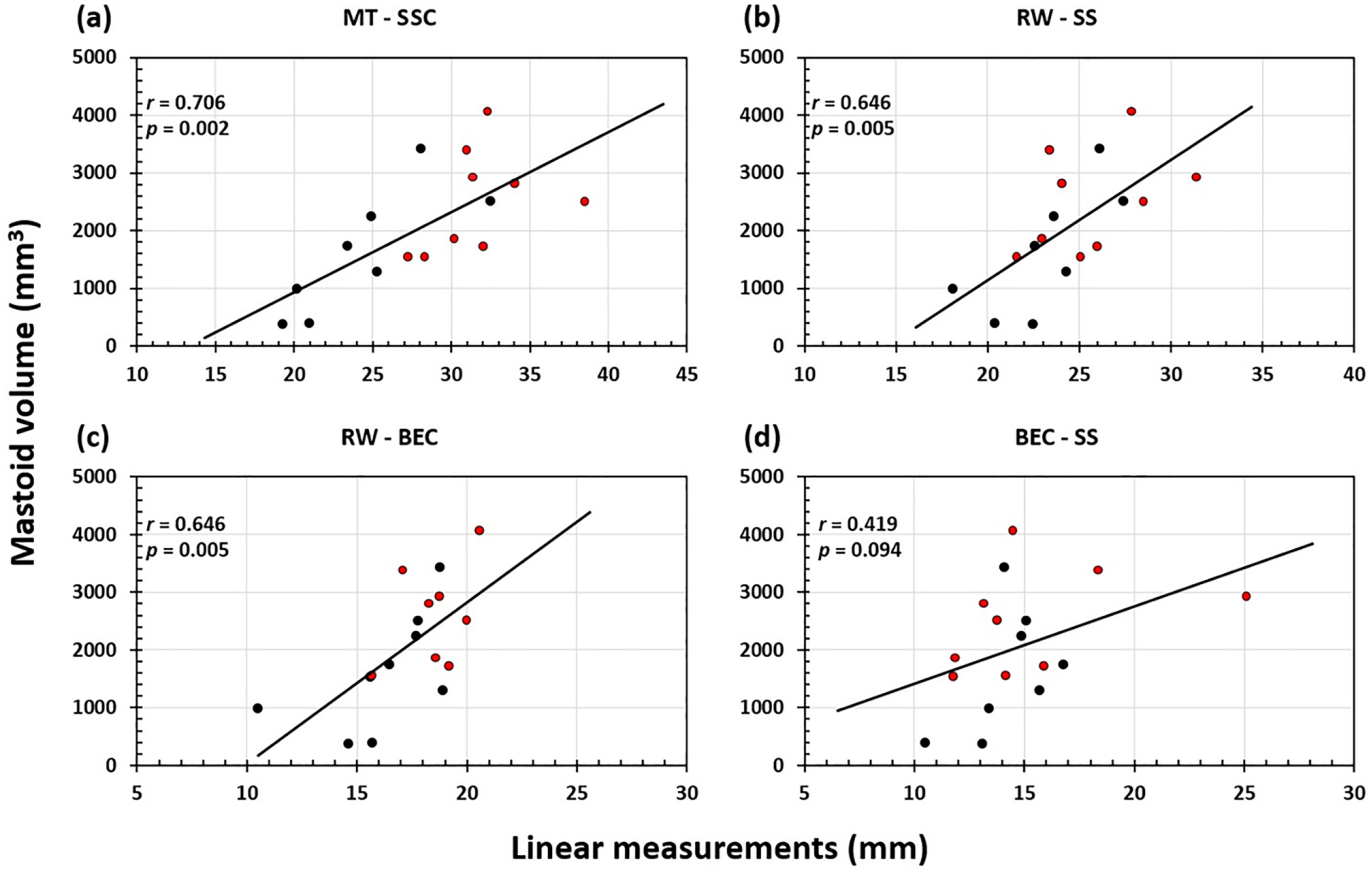
Correlation plots between linear measurements (MT-SSC **a**, RW-SS **b**, RW-BEC **c**, BEC-SS **d**) and mastoid volume. Black datapoints refer to pre-op measurements and the red datapoints refer to post-op measurements.

Plotting the linear measurements between key anatomical points and mastoid volume shows a linear positive correlation (Fig. 5a–d). The correlation between linear measurement and volume were strong between MT-SSC (*r* = 0.706, *p* = 0.002), RW-SS (*r* = 0.646, *p* = 0.005) and RW-BEC (*r* = 0.646, *p* = 0.005). The measurement between RW and BEC did not show any significant correlation (*r* = 0.419, *p* = 0.094).

Patient 1 as shown in Fig. 1a had electrode migration on the right ear and this was suspected to have happened between 2.5 and 3 years after implantation. The patient had normal impedances at 2.5-year follow-up but in the 3-year follow-up the impedance of the basal three electrode contacts had increased beyond the measurable limit and the control CT image showed 6 electrode contacts outside of the cochlea. During the revision surgery, we observed significant bone growth around the electrodes in the middle ear and the mastoid. In rest of the cases migration was not suspected or observed.

## Discussion

Growth of the mastoid with age in relation to CI surgery is an important topic of research, as the coiling of excess electrode lead in the mastoid drilled cavity could be affected by the mastoid growth. Electrode migration is a postoperative complication associated with CI surgery and very few studies have addressed its cause. Vaid et al.^5^ in 2011 discussed for the first time in her case study that the natural growth in mastoid thickness with age could be a reason for electrode extrusion. Our centre had reported earlier on electrode array migration out of the cochlea with a lateral wall electrode type^6^ and that has motivated us to a better understanding on the effects of CI surgery on mastoid growth in CI patients. To the best of our knowledge, this is the first study of this kind.

In the present study, we have shown four novel linear measurements that define the mastoid process and its natural growth with age in CI implanted patients. The linear distance from the RW to SS and BEC captures the overall mastoid as seen in the axial plane. Such measurements are more practical to make from images directly from the PACS software using MPR. The linear distance from the SSC to MT defines in a way the mastoid thickness in the coronal view and the BEC to SS in the axial view. All these linear measurements were found to increase with time as determined between the pre- and the postoperative images. The mastoid volume was also seen to increase with time and with the linear measurements.

Our findings are in line with earlier reports showing the growth in mastoid process over time. Cinamon et al.^4^ in 2009 reported on mastoid air cell growth rate of 1–1.2 cm^2^/year between the age of 1–6 years from the non-CI implanted cases. Almuhawas et al.^11^ in 2020, Zhao et al.^12^ in 2019, Karakas et al.^13^ in 2005, Chatterjee et al.^14^ in 1990 and Vyrekov et al.^15^ in 1977 reported rapid growth in mastoid up to 10 years of age from the non-CI implanted cases. Schilde et al.^16^ in 2017 reported on the temporal bone growth including the mastoid volume in non-implanted patients using three-dimensional (3D) reconstructed models, whereas our approach involved applying regular PACS-MPR that are used in the clinical practice. In comparison to the other methods published so far, the benefit of our method is the definition of a consistent plane for measurements, which enables the measurements between follow-up images to be aligned in same manner as the first image. Schilde et al. reports that the growth rate of the mastoid is 0.76 ml/year in males and 0.53 ml/year in females^16^. In our study, we found that the growth rate of mastoid was on average 239.48 mm^3^/year which is 0.239 ml/year. Lee et al. reported an average growth rate of the mastoid per year of 4.32 ml and 3.31 ml/year in males and females, respectively.

In the present study, the mean preoperative volume was 1.62 ml (0.37–3.72 ml) between the age of 0.92–3.92 years, while the mean postoperative volume was 2.43 ml (1.53–4.06 ml) between the age of 3.25–11.33 years. It is known that the size of the mastoid varies considerably between individuals, so comparing the volume to another study says relatively little about the effects of the CI on the mastoid growth in our study. It seems that the growth rate in the mastoid volume (Table 2) is lower compared to the findings of Schilde et al., except in Patient 1 with electrode migration. The electrode migration was on the right ear with six basal electrodes extruded out of the cochlea and the growth of the mastoid was not enlarged compared to the other cases (Table 2). The measurement between RW and BEC remained unchanged in patient 1 considering measurement error. This was observed at less than one year of age in the preoperative images when the mastoid volume was quite small. The postoperative volume was no bigger than other cases, but the Patient 1 was much younger than other patients at the postoperative time point. Another interesting finding was the change in mastoid volume in Patient 7. The mastoid should reach its final size around the age of puberty. However in this case, the volume did not increase over 7 years and 5 months of follow-up period, despite the patient being close to puberty at the end of follow-up period. It is hard to speculate if the mastoid drilling during the CI surgery may have affected the mastoid growth following the surgery in this case, whereas not in other cases. Due to smaller sample size, we could not draw a conclusion if the mastoid drilling could affect the mastoid growth; thus, studies with larger sample size are needed to investigate these findings.

The mastoid volume measured in this study is in close agreement with the earlier findings and generally endorses the use of the simple measurement techniques applied in this study. The linear distances between RW-SS, RW-BEC and SS-BEC forms the three sides of the triangle in our measurement system. To the best of our knowledge, there are no previous reports on such a uniform method of mastoid measurement using the PACS system that showed a clear correlation between linear measurements and mastoid volume. Our method has the advantage of aligning the images in a standardized format from which the measurements are made. While the proposed method seems promising, a validation of the technique with a larger sample size and reproducibility is required.

## Conclusion

We observed an increase in the mastoid volume and the linear distances between key anatomical points in the mastoid bone comparing the pre-operative and post-operative images over time. Drilling away most of the mastoid air cells for cochlear implantation does not seem to affect the natural growth of the mastoid air cell system in children. Due to the small sample size, it remains inconclusive, whether mastoid growth is associated with electrode migration and therefore warrants more research.

## Data Availability

All data generated or analysed during this study are included in this published article.
